# Laser Induced Damage of Potassium Dihydrogen Phosphate (KDP) Optical Crystal Machined by Water Dissolution Ultra-Precision Polishing Method

**DOI:** 10.3390/ma11030419

**Published:** 2018-03-13

**Authors:** Yuchuan Chen, Hang Gao, Xu Wang, Dongming Guo, Ziyuan Liu

**Affiliations:** Key Laboratory for Precision and Non-traditional Machining Technology of Ministry of Education, Dalian University of Technology, Dalian 116024, China; pacocyc@163.com (Y.C.); wx382935877@163.com (X.W.); guodm@dlut.edu.cn (D.G.); lzy5307827@126.com (Z.L.)

**Keywords:** laser damage, KDP crystal, ultra-precision machining, water dissolution polishing

## Abstract

Laser induced damage threshold (LIDT) is an important optical indicator for nonlinear Potassium Dihydrogen Phosphate (KDP) crystal used in high power laser systems. In this study, KDP optical crystals are initially machined with single point diamond turning (SPDT), followed by water dissolution ultra-precision polishing (WDUP) and then tested with 355 nm nanosecond pulsed-lasers. Power spectral density (PSD) analysis shows that WDUP process eliminates the laser-detrimental spatial frequencies band of micro-waviness on SPDT machined surface and consequently decreases its modulation effect on the laser beams. The laser test results show that LIDT of WDUP machined crystal improves and its stability has a significant increase by 72.1% compared with that of SPDT. Moreover, a subsequent ultrasonic assisted solvent cleaning process is suggested to have a positive effect on the laser performance of machined KDP crystal. Damage crater investigation indicates that the damage morphologies exhibit highly thermal explosion features of melted cores and brittle fractures of periphery material, which can be described with the classic thermal explosion model. The comparison result demonstrates that damage mechanisms for SPDT and WDUP machined crystal are the same and WDUP process reveals the real bulk laser resistance of KDP optical crystal by removing the micro-waviness and subsurface damage on SPDT machined surface. This improvement of WDUP method makes the LIDT more accurate and will be beneficial to the laser performance of KDP crystal.

## 1. Introduction

Potassium Dihydrogen Phosphate (KDP) crystal is currently one of the important and irreplaceable optical components used in high power laser systems for inertial confinement fusion (ICF) research [1,2,3]. However, its laser damage resistance becomes the key factor that severely restricts the output performance of the lasers [4] and this is an urgent technological issue to be resolved in the engineering application.

Laser induced damage mechanisms in optical materials are classified into two categories: extrinsic mechanisms associated with absorbing inclusions and intrinsic mechanisms, which are inherent to intrinsic processes in defect-free materials [5]. For nanosecond scale pulsed-laser radiation widely used in fusion-scale experiment, laser induced damage in KDP is usually believed to be initiated at nanometer absorbing centers in the bulk of the material during crystal growth [6,7,8] and the small defects introduced during the technological fabrication processes [9,10,11]. They either absorb energy and then conduct it to the surrounding host material leading to rapid heating and ionization (thermal explosion theory) [12], or create surface modulation effect on lasers and transmission wavefront distortion [13] and finally cause laser damage of optical materials. Therefore, it is of great significance to improve the laser induced damage threshold (LIDT) by reducing and removing damage precursors. The extrinsic mechanisms subjected to manufacturing technologies can be reduced to improve LIDT of KDP crystal [14]. Efforts have been made to achieve flawless surface for KDP crystal with high laser resistance performance. Traditional ultra-precision grinding and polishing methods developed for optical manufacturing usually use specially prepared grinding or lapping tools with corresponding slurry or fluid [15,16]. Then the obtained surface roughness reaches nanometer level and LIDT improves. Novel optical polishing techniques are adequately proven and considered state-of-the-art technologies for KDP finishing because the processed elements possess excellent laser damage performance, low subsurface imperfection and high surface quality [17,18,19,20]. However, the magnetorheological finishing (MRF) [21,22] and water dissolution ultra-precision polishing (WDUP) [23,24,25] have been frequently studied recently, for they can remove the single point diamond turning (SPDT) machined marks left on the crystal surface. Polishing, aimed at removing the diamond tool turned waviness, is considered a feasible way to increase the LIDT of KDP optical components. Especially those processing technologies using the nonaqueous carrier fluid with water content that utilizes the kinetic energy to achieve material removal [26,27]. 

Many scholars have reported on MRF of KDP and its advantages over other machining methods on LIDT. However, there are relatively few studies devoted to the LIDT investigation of WDUP polished crystals. Therefore, in this study, laser damage experiments are carried out on KDP crystals machined with WDUP method. Damage morphologies are investigated thoroughly and the damage mechanisms are discussed. The influence of WDUP process on the LIDT of KDP optical crystal is analyzed. The findings in this work might provide a valuable insight for further study.

## 2. Experimental

### 2.1. Samples and Processing

**Figure 1:**
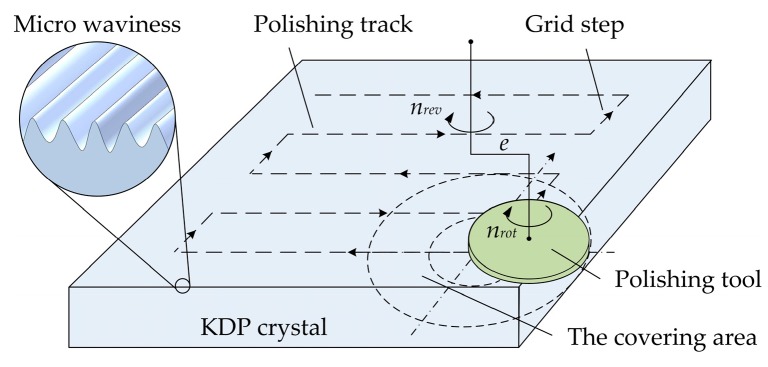
Schematic of the ultra-precision polishing principle with CCOS technology for removing the diamond turned micro-waviness.

The KDP samples used in the experiment are tripler crystals with a size of 30 × 30 × 10 mm^3^ which are then machined by SPDT to get the ultra-precision surface. During the diamond turning process, the spindle speed is 390 r/min, feed velocity is 100 μm/s, cutting depth is 15 μm and the diamond tool radius is 5 mm. In order to compare surface quality and laser induced damage of machined surfaces by SPDT and WDUP respectively, the diamond turned samples are numbered and grouped, part of which are polished with a subsequent WDUP process and other groups are prepared for testing the laser damage threshold of SPDT. The polishing principle for removing the diamond turned micro-waviness is illustrated in Figure 1 [27]. The polishing pad is IC 1000 type (ROHM & HAAS, Philadelphia, PA, USA) made from polyurethane with groove to store the polishing fluid. During the polishing process, the small polishing tool with a rotation spindle (speed *n_rot_* = 110 r/min) and a revolution spindle (speed *n_rev_* = 110 r/min, with an eccentricity *e* = 3 mm) performs a planetary motion. Polishing pressure is 34 kPa and feed velocity is 50 mm/min. The polishing covering area moves along the predesigned path (grid step is 5 mm) by computer controlled optical surfacing (CCOS) technology and finally achieves entire ultra-precision surface. Polished samples are immediately wiped off of polishing fluid and some of them are further cleaned with specific solvents for the preparation of laser test. The diamond turning experiment and the polishing experiment are conducted under the class-1000 clean laboratory environment at 25 °C.

### 2.2. Laser Damage Test

**Figure 2:**
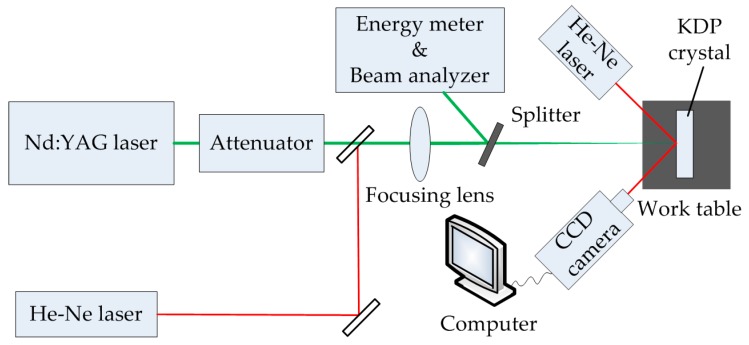
Schematic of experimental setup for measuring laser induced damage of Potassium Dihydrogen Phosphate (KDP) crystals.

The schematic of laser system built up for testing SPDT and WDUP processed KDP crystals is shown in Figure 2. The measuring method adopted in the experiment was 1 on 1 test [28] and 10 sites were arranged for each laser fluence. A mode-locked Nd:YAG laser working at third-harmonic (Continuum PL8000, wavelength 355 nm, 1 Hz repetition rate) (Continuum, Boston, MA, USA) is used for the laser initiation. The laser beam has a Gaussian temporal with 6.8 ns pulse duration. The incident angle is 0° and the bean is focused by optical lens to the sample surface. The working distance is 2 m. The measured effective spot area of the laser beam is 0.22 mm^2^. One He-Ne laser is positioned as the beam collimator in the laser path. Another He-Ne laser is also focused onto the KDP sample to illuminate any resulting damage in assisting the observation of the damage sites. The real-time damage information is displayed on the computer screen through a CCD camera. The laser test experiments were carried out at 25 °C and 43% relative humidity (RH).

## 3. Results and Discussion

### 3.1. Surface Quality Analysis

**Figure 3:**
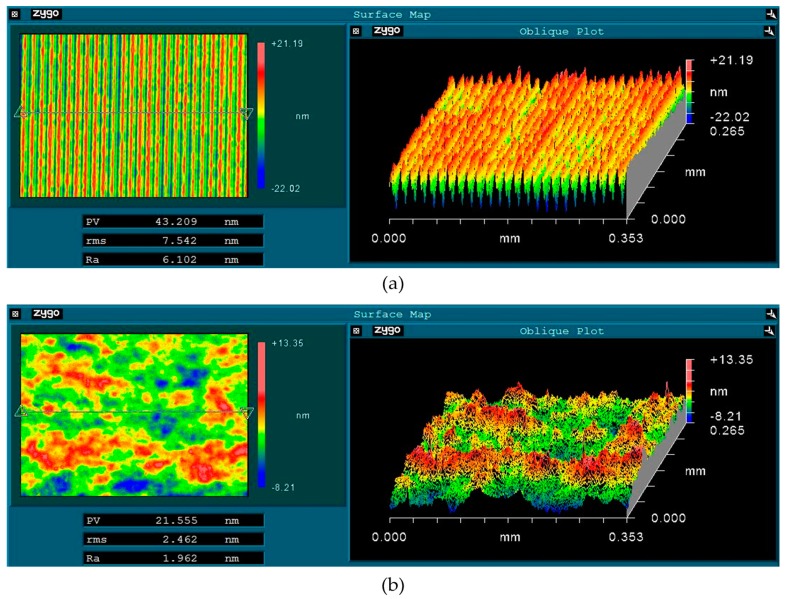
Topography of KDP crystal surface (**a**) Machined by single point diamond turning (SPDT) (PV 43.209 nm, RMS 7.542 nm); (**b**) Machined by water dissolution ultra-precision polishing (WDUP) (PV 21.555 nm, RMS 2.462 nm).

Surface quality improvement is closely related to better laser damage performance for KDP crystals. Therefore, the surface topography of the processed KDP crystal surface is measured with a white light interferometer (Newview 5022, ZYGO, Berwyn, PA, USA) over an area of 0.353 × 0.265 mm^2^. For a typical SPDT machined surface, it can be observed that a micro textured surface with uniform undulation of micro-waviness along a certain direction (shown in Figure 3a), which is caused by the machine feeding pitches of the single point diamond tool. The measured amplitude of the micro grooves ranges from 30 nm to 35 nm and has a period of approximately 15 μm. The surface achieved is a super smooth surface with 7.542 nm root-mean-square (RMS) roughness and 43.209 nm peak-to-valley (PV) value. A KDP crystal surface processed by WDUP presents a flat and smooth appearance without any machining marks (shown in Figure 3b). This is because the selective material removal mechanism of WDUP method, which is well illustrated in Reference [29]. Combined with CCOS technology, the whole material removal process is precisely controlled to achieve the ultra-precision machining of KDP optical crystal. The obtained surface has a 2.462 nm RMS and 21.555 nm PV, which indicated a significant improvement in the surface quality of the crystal. The average surface RMS roughness for processed KDP samples (shown in Table 1) proves the improvement in surface quality for WDUP after SPDT.

In order to make clear of this, power spectral density (PSD) analysis are done on the machined surface according to discrete form of two-dimensional (2D) PSD equations [30],

(1) $$PSD\left(f_{x}f_{y}\right)=\frac{{\left(\Delta x\right)}^{2}}{MN}|\overset{N-1}{\underset{n=0}{\sum }}\overset{M-1}{\underset{m=0}{\sum }}Z\left(n,m\right)\exp\left(-i2\pi \left(my/M+nx/N\right)\right){|}^{2}$$

or in one-dimensional (1D) case,

(2) $$PSD\left(f_{x}f_{y}\right)=\frac{\Delta x}{N}|\overset{N-1}{\underset{n=0}{\sum }}Z\left(n\right)\exp\left(-i2\pi nx/N\right)){|}^{2}$$

where, *Z*(*n*, *m*) is the height of the surface profile over a discrete array of *N* by *M*, Δx is sampling step, *f_x_* and *f_y_* are the spatial frequency along X and Y directions and $f_{x}=n/\left(N\Delta x\right)$, $f_{y}=m/\left(M\Delta x\right)$, −N/2≤x≤N/2, −M/2≤y≤M/2.

**Figure 4:**
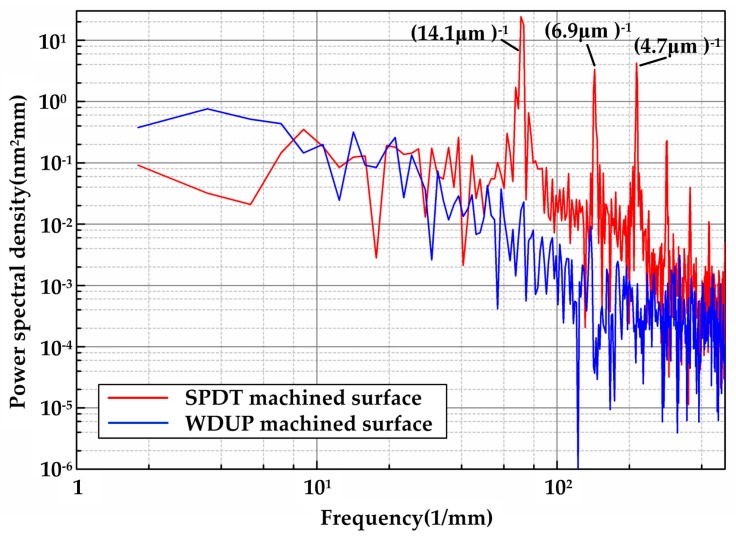
1D-PSD comparison of Single Point Diamond Turning (SPDT) and water dissolution ultra-precision polishing (WDUP) machined crystal surface.

**Figure 5:**
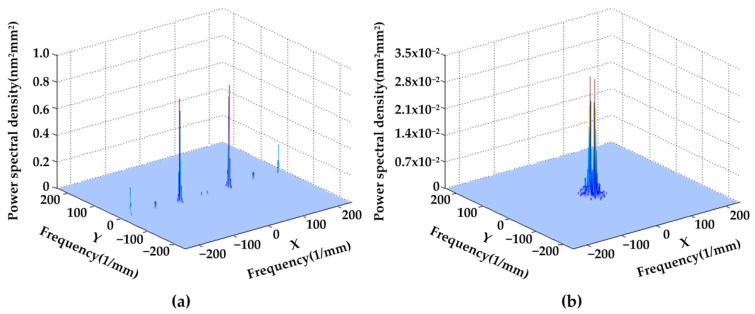
2D-PSD comparison (**a**) SPDT machined surface (distribution along X frequency with maximum value of 0.7 nm^2^mm^2^) (**b**) WDUP machined surface (higher concentration of low spatial frequency with maximum value of 2.8 × 10^−2^ nm^2^mm^2^).

For high power laser facilities required crystals, the wave front quality over surface spatial frequencies is frequently stressed and strictly specified [31]. PSD, simply identified as the square value of the phase noise amplitude over a certain spatial frequency (mm^−1^), describes the “micro-waviness” distribution of a surface morphology. Figure 4 shows PSD curves of KDP surface detached from SPDT and WDUP, respectively. For 1D PSD analysis, the data is taken from an arbitrary line crossing the surface since the polished surface is flat without directional features. While it is taken in the direction perpendicular to the turned micro-waviness for SPDT machined crystal surface. As it can be seen, the highest peak at the position of (14.1 μm)^−1^ is perfectly aligned with the period of micro waviness (approximately 15 μm). The magnitude of WDUP machined surface PSD ranging from 4.5 mm^−1^ to 500 mm^−1^ does not exceed that of the SPDT machined surface and is evidently much lower. The peaks reflecting the features of SPDT are removed after WDUP. For 2D PSD shown in Figure 5, it is very clear that the spatial frequency is mainly concentrated on some certain bands along X direction on the SPDT machined surface (Figure 5a) and these detrimental frequencies are corresponding to the cutting periodic ripples. After WDUP, the middle and high spatial frequency portions of SPDT are fully removed (Figure 5b) and the polished surface presents less surface error with higher concentration of PSD and lower PSD value (2.8 × 10^−2^ nm^2^mm^2^ of WDUP to 0.7 nm^2^mm^2^ of SPDT). 

From the above analysis, it can be concluded that WDUP removes detrimental micro-waviness and its spatial frequency bands are caused by SPDT on a machined surface, which greatly improves the surface integrity of KDP optical crystal. Most important is that the reduction of the spatial frequency consequently mitigates the modulation effect of the surface on laser beams [14], which will be beneficial to the laser resistance performance of optical elements and make the LIDT of WDUP finished KDP optical crystal more accurate.

### 3.2. Laser Induced Damage Threshold Analysis

**Figure 6:**
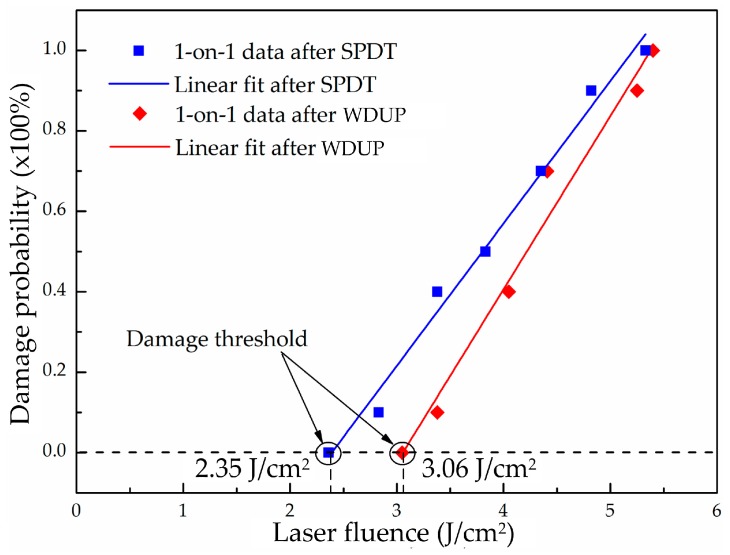
Laser induced damage threshold (LIDT) calculation of the tested KDP crystals.

The calculations of the laser damage threshold are based on 1-on-1 test and the linear fitted zero damage probability is taken as the LIDT at 355 nm wavelength for processed KDP crystal. Figure 6 shows the LITD calculation of the tested KDP crystals after SPDT and WDUP. As can be seen from the figure, the LIDT of a SPDT machined KDP sample is 2.35 J/cm^2^ and that obtained from a WDUP machined sample is 3.06 J/cm^2^, which shows a slight improvement of the damage threshold for ultra-precision polishing over diamond turning on one tested group. However, a laser damage test is a probability event and a number of KDP samples are exposed to laser irradiation in this experiment. LIDT test results of all KDP samples after SPDT and WDUP are shown in Table 2. Also LIDT results of additional cleaning experiment after WUDP are added to Table 2 in order to validate the positive effect of subsequent cleaning process on LIDT performance of KDP crystal. The ultrasonic assisted solvent cleaning experiments are conducted on other KDP crystal samples and the cleaning principle is similar compatible theory of organic solvents, which is well illustrated according to reference [32].

**Figure 7:**
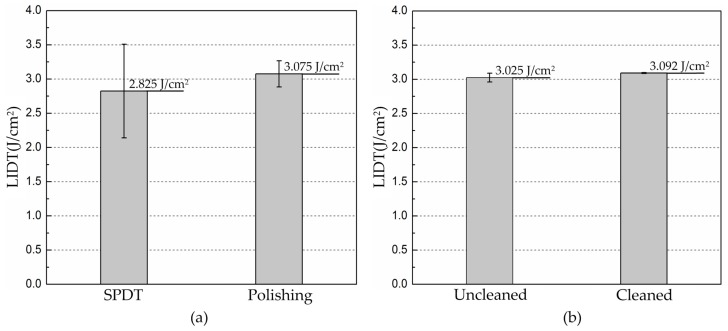
Comparison of LIDT and its stability (**a**) After SPDT and WDUP (**b**) Uncleaned and cleaned after WUDP.

The statistical results indicate that post ultra-precision polishing process after diamond turning is beneficial to the laser performance of KDP optical crystal. Figure 7 plots the experimental results of LIDT and its distribution comparisons. Since the two manufacturing methods are ultra-precision machining, the LIDTs in the experiment vary little and that of WDUP improves slightly on average (from 2.825 J/cm^2^ for SPDT to 3.075 J/cm^2^ for WDUP). But its standard deviation shows a significantly increase by 72.1%, as predicted. The results in Figure 7b clearly illustrates that cleaning is beneficial to the LIDT (from 3.025 J/cm^2^ to 3.092 J/cm^2^) as well as its stability (a significant improvement of 89%). We suppose that the subsequent cleaning process helps to eliminate KDP surface residue and near-surface particles and contaminations which could become damage precursors. However, the KDP samples used in the study are the unconditioned as-grown crystal and the primary damage in the bulk of the material are limited to a low level at 3*ω* [33]. Therefore, the results of further laser damage tests will be investigated and reported in the future.

### 3.3. Laser Damage Sites Observation

**Figure 8:**
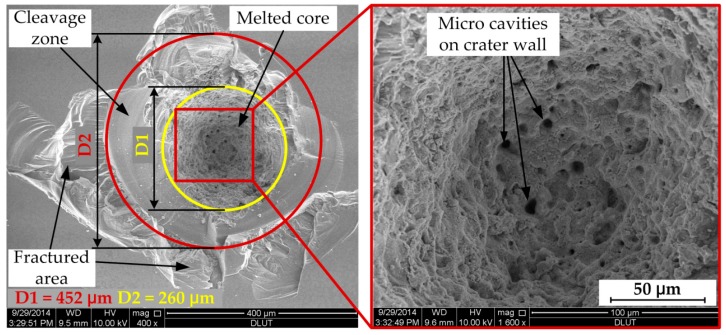
Typical appearance of damage morphology on processed KDP surface.

Microscopic observation of the processed KDP surface damage is carried out with a scanning electron microscope (SEM, Q45, FEI, Hillsboro, OR, USA) under low pressure mode and voltage of 10 kV or 12.5 kV. Figure 8 shows the typical morphology of a damaged site under a single high-fluence laser pulse test. It presents a cone-shaped damage crater with a melted core region and fractured periphery, whose size is very large to approximately 500 μm in diameter. As it can be seen and is marked in the picture, the damage morphology is apparently divided into three regions according to their formation mechanisms. The core area of damage crater (diameter about 260 μm) has characteristics of circular lateral symmetry, porous internal walls of melted material flow and resolidification. These features evidently point out the heating of precursors reaches the maximum value and critical temperature at the absorber/matrix interface, leading to the effective conversion of the defect-surrounding bulk material into absorbing medium, rapid heating, laser ionization and material ablation. The porous crater wall with micrometer caves is supposed to result from the surrounding nano-sized precursors absorbing and dissipating energy from the melting core [34]. However, the periphery morphology exhibits features of a random lateral shape with fracture and spallation caused by material failure. While they can still be classified into two different formation categories, the area between two circles shows the smooth crystal cleavage plane detached by a strong shock wave force from the adjacent melting core and the outermost zone is common fractured material and cracks stretching outward. Because KDP crystal is brittle and highly anisotropic, the direction of fracture is random and unpredictable.

**Figure 9:**
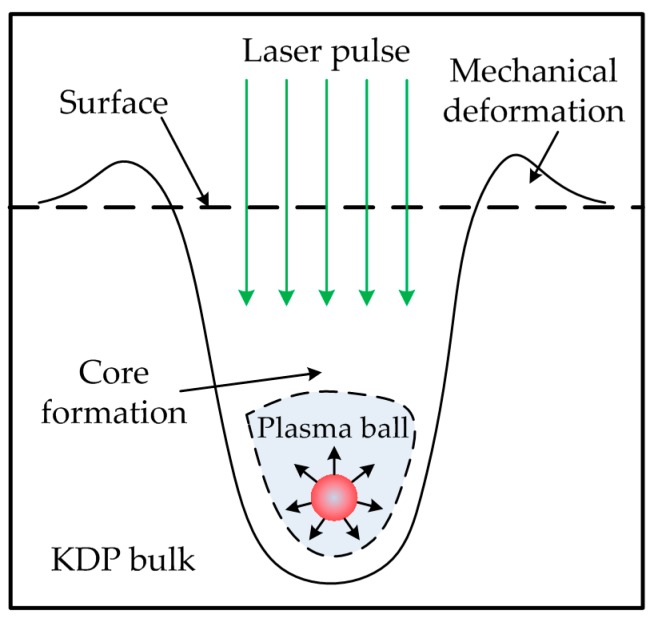
Thermal explosion mechanism of plasma-ball formation around an absorber.

The damage morphology formation mechanism described above exhibits highly thermal explosion features of high power pulsed-laser dielectric material interactions. The crater-formation scenario is believed to be explained by the classical thermal explosion model as depicted in Figure 9 [35] and described by an inclusion-initiated thermal-diffusion model in following equations [11].

(3) $$\frac{\partial T}{\partial t}=D\nabla^{2}T$$

with boundary conditions at *r* = *a*

(4) $$\alpha I\left(t\right)=-4k{\left(\frac{\partial T}{\partial r}\right)}_{\tau -a}+\frac{4\rho Ca}{3}{\left(\frac{\partial T}{\partial r}\right)}_{\tau -a}$$

where *r* is the distance to the particle center, *a* is the particle radius, *t* is time, *I* is the laser intensity, *D* and *k* are thermal diffusivity and thermal conductivity of the matrix, respectively, *α*, *ρ* and *C* are, respectively, absorptivity, density and heat capacity of inclusion, *T* is the temperature.

**Figure 10:**
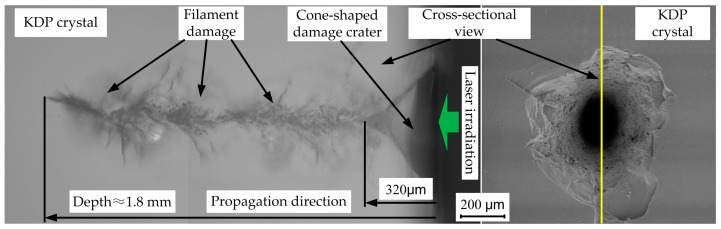
Cross-sectional view of a typical damage crater on processed KDP surface.

In order to investigate the complex damage morphology of the craters, a cross-sectional picture of a typical damage site on processed KDP surface under 355 nm laser irradiation is shown in Figure 10. From top view in the entrance surface, the damage morphology just shows the same as that described above by thermal explosion theory. However, side view of the damage crater reveals the complex detailed information of the overall crater structure. The damage crater has a cone shape in entrance surface caused by the thermal eruption outward and a long “tail” of filament damage along the laser propagation path. The cone shape ends at a depth of 320 μm and the filament damage extends far deeper (to millimeter scale) in the bulk crystal, which indicates that laser caused damage can penetrate deep into KDP crystal. Filament damages are collection of melted cores on the central path which indicates a liquid or vapor phase in the energy deposition stage and the material failure in the vicinity which is caused by laser ionization of damage precursors when laser passes through some critical depth. This type of bulk damage is believed to be the consequence of two damage mechanisms, self-focusing effect and defects-initiated absorption [36,37]. This damage crater formation scenario suggests that the physical damage process which dominants energy deposition and destructive heating of highly absorbing material due to thermal diffusion of laser fluence is the main damage mechanism. The analysis of cross-sectional damage morphology helps to understand the inner damage in the KDP crystal bulk. 

**Figure 11:**
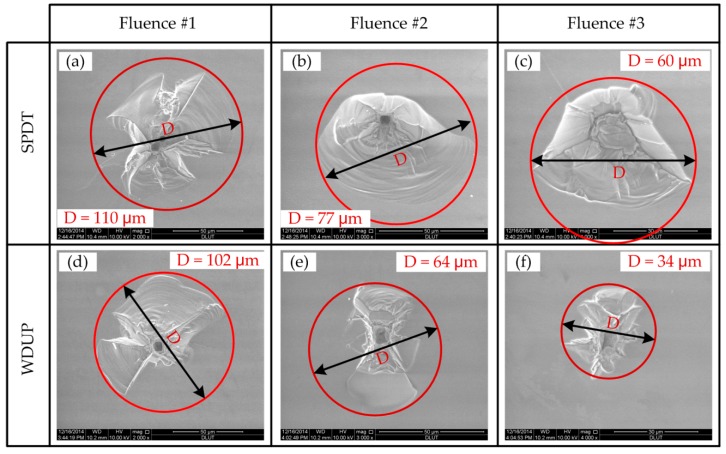
Damage morphologies of SPDT and WDUP machined crystal surface (Fluence#1 > Fluence#2 > Fluence#3).

Figure 11 shows the Scanning Electron Microscopy (SEM) pictures of damage morphologies on the KDP surface processed after SPDT and WDUP under different laser fluence at 355 nm. Characteristics of damage sites are the appearance of crater-shaped melted core with fractured surroundings and the fractured area pointing to the exploding breakage of the materials takes the large proportion. In terms of crater formation and damage mechanism, experimental results indicate no obvious difference between surfaces finished by these two precision mechanical manufacturing processes. However, the size of the damage crater and the extent of the cracks vary greatly under the same laser fluence level. Under higher fluence (Fluence#1 in Figure 11), the crater sizes after SPDT and WDUP are almost the same. With the decrease of the laser fluence, the damage sites on the WDUP machined surface show dramatic reduction in size (from about 100 μm to 30 μm) while those of the SPDT processed surface do not shrink that much in size (from about 110 μm to 60 μm). This demonstrates the increased capability of the laser resistance property of KDP crystal after WDUP. 

**Figure 12:**
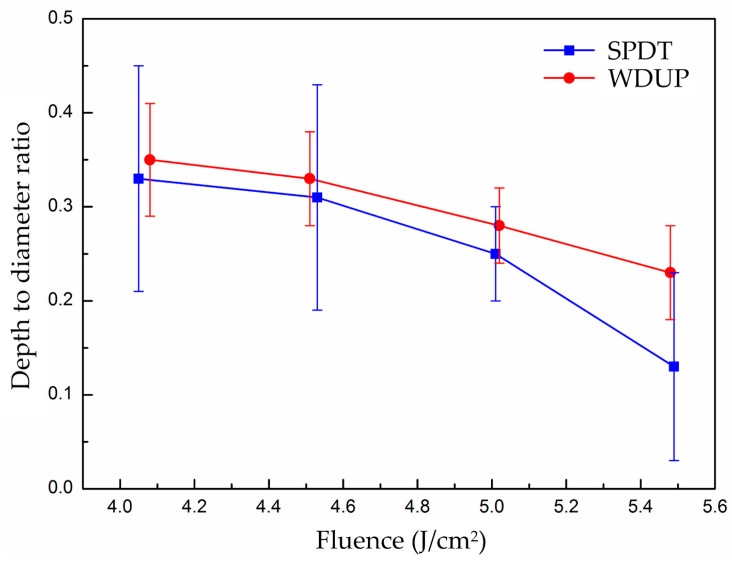
Depth to diameter ratio of damage crater versus laser fluence of KDP crystal processed with SPDT and WDUP.

The subsequent WDUP process must remove some detrimental damage factors on the SPDT machined surface. On the other hand, the main morphology of the fractured part is a cleavage plane caused by the spallation of crystal material on the SPDT machined surface. However, signs of cracks resulting from plastic deformation or material failure take the dominant path in the periphery of the WDUP crystal surface. The difference in the fractured part formation is probably associated with that the WDUP process removes the subsurface damage and then emerged KDP damage-free material bears the destructive force when exposed to laser irradiation. For SPDT machined surface, the micro-cracks and defects layer beneath the micro-waviness weakens the material strength and makes it vulnerable to external shock wave forces, which can account for the spallation of cleaved planes. The ratio of depth to diameter for damage craters created at each laser fluence on SPDT and WDUP machined surfaces are plotted (shown in Figure 12) to further understand the damage formation. The ratios for both methods are seen to decrease with the increase of laser fluence and the ratio over full range fluence is a little lower but with larger fluctuations for SPDT machined surfaces than those of WDUP, which indicates larger damage crater formation for SPDT machined surface under an equivalent depth, especially for the very high laser fluence. The result agrees with the above analysis that SPDT machined surfaces with subsurface damage easily cause cracks and material failure. 

The laser damage processes and crater morphologies for these two scenarios clearly demonstrate that laser heating and material structural deformation are coupled to each other and then contribute to the laser induced damage of KDP crystal. The laser damage mechanisms of SPDT and WDUP machined surfaces are the same. The finding also suggests that laser induced damage for WDUP polished surfaces is closer to the damage threshold of the ideal bulk KDP crystal. This is believed to be associated with the reduction of detrimental micro-waviness as well as the subsurface damage [27] on crystal surfaces after polishing succeeding the SPDT process. This result coincides with the hypothesis that subsurface structural damage introduced by the ultra-precision machining technology is the underlying cause of lower LIDT of KDP optical crystal [38]. The reduction of micro-waviness and the removal of shallow subsurface damage after WDUP improves the LIDT and the stability of KDP optical components. This is very important for optical material finishing.

## 4. Conclusions

KDP crystals are processed to an ultra-precision surface with SPDT and WDUP methods. Then, a laser induced damage performance comparison of the machined KDP crystals at 355 nm is conducted using a 1-on-1 test. PSD analysis shows that the WDUP process eliminates the laser-detrimental spatial frequency bands of micro-waviness on SPDT machined surfaces. Furthermore, the LIDT is analyzed and damage morphologies of the craters on the machined surface are studied. The results show that the WDUP method has a positive influence on the LIDT of unconditioned KDP crystal with a significant increase of 72.1% in its stability and a subsequent ultrasonic assisted solvent cleaning process is believed to be beneficial to the LIDT performance of WDUP polished KDP crystal. The laser damage mechanisms for SPDT and WDUP machined crystal are the same and can be described with the classic thermal explosion model. The damage formation is a brittle fracture in the periphery with a melted core caused by laser absorption of inclusions. The WDUP method can effectively remove the micro-waviness of tool marks and subsurface damage left by the SPDT process, thus revealing the real laser resistance surface without introducing additional damage, which improves the surface quality. The improvement of the surface quality decreases the modulation effect of the spatial frequency on the laser beams and makes the LIDT more accurate and consequently increases the laser resistance performance of KDP optical crystal.

## Figures and Tables

**Table 1:** Averaged surface Root Mean Square (RMS) roughness of processed KDP samples (6 crystals in each group).

Processing Method	Surface Feature	RMS Roughness/nm
SPDT	Micro-waviness	7.442
WDUP	Smooth	2.349

**Table 2:** LIDT test results of KDP samples with different processing methods (6 crystals in each group, test environment: temperature 25 °C, humidity 43% RH).

Processing Method	Laser Induced Damage Threshold (LIDT) (J/cm^2^)
SPDT	2.825 ± 0.684
WDUP	3.075 ± 0.191
WDUP (uncleaned)	3.025 ± 0.064
WDUP (cleaned)	3.092 ± 0.007
